# A computational model of invasive aspergillosis in the lung and the role of iron

**DOI:** 10.1186/s12918-016-0275-2

**Published:** 2016-04-21

**Authors:** Matthew Oremland, Kathryn R. Michels, Alexandra M. Bettina, Chris Lawrence, Borna Mehrad, Reinhard Laubenbacher

**Affiliations:** Mathematical Biosciences Institute, Ohio State University, 1735 Neil Ave, Columbus OH, USA; University of Virginia, Pulmonary and Critical Care Medicine, Charlottesville VA, USA; Virginia Bioinformatics Institute, Virginia Tech, 1015 Life Science Circle, Blacksburg VA, USA; Center for Quantitative Medicine, University of Connecticut Health Center, 236 Farmington Ave, Farmington CT, USA; Jackson Laboratory for Genomic Medicine, 236 Farmington Ave, Farmington CT, USA

**Keywords:** Invasive aspergillosis, Agent-based model, Lung, Iron

## Abstract

**Background:**

Invasive aspergillosis is a severe infection of immunocompromised hosts, caused by the inhalation of the spores of the ubiquitous environmental molds of the *Aspergillus* genus. The innate immune response in this infection entails a series of complex and inter-related interactions between multiple recruited and resident cell populations with each other and with the fungal cell; in particular, iron is critical for fungal growth.

**Results:**

A computational model of invasive aspergillosis is presented here; the model can be used as a rational hypothesis-generating tool to investigate host responses to this infection. Using a combination of laboratory data and published literature, an *in silico* model of a section of lung tissue was generated that includes an alveolar duct, adjacent capillaries, and surrounding lung parenchyma. The three-dimensional agent-based model integrates temporal events in fungal cells, epithelial cells, monocytes, and neutrophils after inhalation of spores with cellular dynamics at the tissue level, comprising part of the innate immune response. Iron levels in the blood and tissue play a key role in the fungus’ ability to grow, and the model includes iron recruitment and consumption by the different types of cells included. Parameter sensitivity analysis suggests the model is robust with respect to unvalidated parameters, and thus is a viable tool for an *in silico* investigation of invasive aspergillosis.

**Conclusions:**

Using laboratory data from a mouse model of invasive aspergillosis in the context of transient neutropenia as validation, the model predicted qualitatively similar time course changes in fungal burden, monocyte and neutrophil populations, and tissue iron levels. This model lays the groundwork for a multi-scale dynamic mathematical model of the immune response to *Aspergillus* species.

**Electronic supplementary material:**

The online version of this article (doi:10.1186/s12918-016-0275-2) contains supplementary material, which is available to authorized users.

## Background

Invasive aspergillosis represents a major and growing health problem in the U.S. and around the world. The growing population of immunocompromised patients, including those with haematologic malignancies, and stem cell- or solid organ-transplant recipients are at highest risk for this disease [1]. In addition to conventionally immunosuppressed patients, other large populations are also at risk of this infection, including individuals with fibrocavitary tuberculosis in developing countries who develop chronic invasive aspergillosis as a secondary infection [2]; it is estimated that in 2007, 372,000 of the 7.7 million new cases of pulmonary tuberculosis world-wide also developed chronic pulmonary aspergillosis [3]. The introduction of new antifungal drugs during the last decade, principally azole-based compounds capitalizing on new insights into the molecular structure of the fungal cell wall, has dramatically improved disease outcomes, but mortality rates remain approximately 30 % in recent surveys [1, 4]. In addition, increased resistance to these new drugs [5] raises the specter of a ‘perfect storm,’ as it has been called in [3], combining a rapidly growing patient population with a diminished repertoire of treatment options.

A substantial body of literature supports the critical role of iron homeostasis in *Aspergillus* biology. *Aspergillus* species adapt to iron-limited environments by activating a system of intracellular and secreted siderophores that scavenge iron from the environment and store it. In in vitro studies, *Aspergillus* siderophores remove iron from transferrin in human serum [6] and impair macrophage iron uptake [7]; conversely, neutrophil lactoferrin inhibits *Aspergillus* conidial growth by sequestering extracellular iron [8]. In animal models, mutant *Aspergillus* species with defective siderophore systems are avirulent [9], and therapeutic iron chelation has an additive benefit to antifungal antibiotics [10]. These mechanisms appear to be clinically important, since among immunocompromised stem cell transplant patients, clinically unsuspected iron overload is an independent risk factor with invasive aspergillosis [11, 12]. Taken together, these data suggest that the competition for iron is a key component of the pathogenesis of invasive aspergillosis.

The innate immune response to invasive aspergillosis is difficult to study. Interrogating dynamic cellular and molecular networks in a human host is, in most cases, impossible. In the study of the innate immune response to *Aspergillus* a number of in vitro and in vivo approaches have been used successfully. These include the in vitro interaction of *Aspergillus* with leukocytes and epithelial cells [13, 14]. In addition, animal models have been a valuable tool to investigate the complexities of cell-cell interactions and inflammatory pathways in a realistic system. These complementary approaches have led to recognition of neutrophils, macrophages, dendritic cells, and lung epithelial cells as key early players in host response to *Aspergillus* species [15, 16]. To date, the focus of the search for new therapeutics has been largely on fungal targets. But more recent promising efforts have looked to the host, in particular host immunity [17, 18]. However, a full exploration of the possibilities for anti-fungal therapeutics targeted at the host requires a better understanding of the innate host response. The complexity of the dynamic regulatory molecular networks and the multi-scale nature of the innate immune response strongly suggest taking a systems biology approach [19], as done in, e.g., [20, 21]. Here, we present a first step toward a multi-scale systems biology model of invasive aspergillosis in the lung, focused on the role of iron. In particular, we present the tissue level component of the model, validated with in vivo data from a mouse model of invasive aspergillosis.

### Related work

Agent-based models (ABMs) are particularly well-suited for capturing the inherent heterogeneity of the immune system; an overview of such models is discussed in [22]; a more focused review of host-pathogen ABMs is presented in [23]. In particular, the complexity of the lung physiology and its effect on dynamic interactions has been established [24], and specific interactions have been the focus of studies on intracellular regulatory networks [21] and the respiratory response to therapeutic interventions [25]. A host-fungus interaction model incorporating data into clinically actionable therapeutic intervention in the case of invasive aspergillosis is presented in [26]. The model presented here incorporates several parameter values and mechanistic behaviors from these models (see Additional files 1 and 2).

The literature indicates the importance of neutrophils and macrophages in the immune response to invasive aspergillosis. The critical importance of neutrophil involvement is detailed in [27], where chemotaxis is found to be the best strategy by which neutrophils find *A. fumigatus* conidia. This study also highlights the importance of pathogen distribution and spatial scale as critical factors, both of which have been incorporated into our model. Another recent ABM described the chemotactic recruitment of macrophages by epithelial cells [28, 29], focusing on the effectiveness of chemotaxis in the role of macrophage response to *A. fumigatus*. This model incorporates specific spatial structure as well as respiratory effects, and provides data on macrophage recruitment time and mechanisms of chemokine diffusion. Our model incorporates both macrophages and neutrophils, using chemokine diffusion and chemotaxis in a spatially heterogeneous domain to investigate the immune response to invasive aspergillosis over time. In addition, we introduce iron as a key factor in the survival of the fungus. ABMs have been used to study the lung in a variety of other contexts as well, including cancer [30], metastasis [31], fibrosis [32], and pneumococcal infection [33]. Studies examining granuloma formation in tuberculosis infection have used ABMs of the lung to investigate chemokine diffusion [34] and pharmacokinetic/pharmacodynamic modeling of antibiotics [35].

There are many software packages for simulation of the immune system in various capacities. These include packages such as SIMMUNE [36], the Basic Immune Simulator [37], SIMISYS [38], and C-ImmSim [39–41]. These packages are general-purpose immune system simulators that, while effective and useful for their intended purposes, are ultimately not appropriate for our purposes.

## Methods

### Animals and in vivo procedures

Female wildtype C57Bl/6 mice were purchased from Jackson Laboratories (Bar Harbor, Maine) and maintained under pathogen-free conditions; experiments were performed in 6- to 10-week old animals. All animal experiments were approved by the Animal Care and Use Committee of University of Virginia.

We used a previously described animal model of invasive aspergillosis in neutropenic hosts [42–44]. Neutrophil depletion was achieved with a single i.p. injection of 80 *μ*g of a monoclonal Ab (Gr-1, clone RB6-8C5) 1 day before an intratracheal challenge with *A. fumigatus*. We have previously reported that this protocol resulted in peripheral blood neutropenia (absolute circulating neutrophil count less than 50 cells/ *μ*L) on days 1 and 3 after injection in both infected and uninfected mice, with a return of peripheral counts to pretreatment levels (>1000 cells/ *μ*L) by day 5 [45, 46] and does not influence the number of other leukocyte subsets in the lung or spleen [44, 47].

### Preparation and administration of *A. fumigatus*

*A. fumigatus* (strain 13073, American Type Culture Collection) conidia were collected in 0.1 % Tween in PBS from 7- to 14- day old cultures on Sabouraud’s dextrose agar plates, filtered through sterile gauze and counted under a hemacytometer. Fungal forms were administered intratracheally in inocula ranging from 2 to 5×10^6^ in 30 *μ*l saline per mouse.

### Identification of leukocyte subsets

At pre-determined time points, animals were euthanized by CO_2_ asphyxiation, the pulmonary vasculature was perfused via the right ventricle with PBS containing 5mM EDTA, whole lungs were removed and single cell suspensions prepared as previously described [43–49]. The following antibodies were used to label cells for flow cytometry (from BD Biosciences, San Jose, CA, or eBiosciences, San Diego, CA): anti-CD11b-allophycocyanin-Cy7 (clone M1/70), anti-CD11c-PE-Cy7 (clone HL3), anti-CD45-peridinin chlorophyll protein (clone 30-F11), anti-Ly-6G-FITC (clone 1A8) and anti-Mac3-PE (clone M3/84). Samples were analyzed on a FACS Canto II instrument using Diva software (BD Biosciences). Neutrophils were identified as CD11b-hi Ly6G+ cells and recruited macrophages as CD11b+ CD11c- Mac3+ cells, as previously described [48, 49]. The absolute number of each leukocyte subset was determined as the product of the percentage of the cell type and the total number of cells in the sample, as determined using an automated cell counter (Countess, Invitrogen, Carlsbad, CA).

### Chitin assay

Since *A. fumigatus* grows as multicellular branching hyphae without forming distinct reproductive units in infected tissues, we used an assay for chitin, a carbohydrate component of hyphal wall that is absent from mammalian tissues and conidia, to quantify the burden of hyphae in infected lungs, as detailed previously [50]. Organ chitin content has been validated as a readout of severity of infection in animal models of invasive aspergillosis by several groups [43, 51–54].

### Measurement of lung iron

Lung iron content was measured as described in [55]. Briefly, lungs were homogenized in 3 ml sterile water and 100 *μ*L of each sample mixed with 100 *μ*L of iron dissociation reagent (equal volumes 20 % trichloroacetic acid 2N HCl in distilled water and 4.5 % KMnO_4_, mixed immediately before use) in duplicate. Samples were incubated for 2 h at 60° C in a chemical fume hood and allowed to cool. Following incubation, 50 *μ*L iron detection reagent (6.5 mM neucoproine, 6.5 mM ferrozine, 1 M L-ascorbic acid and 2.5 M ammonium acetate in distilled water) was added to each sample. After 30 min, samples were centrifuged at 10,000 g for ten minutes and plated on a 96-well plate with an iron standard for Atomic Absorption Spectrometry (Sigma-Aldrich) and optical density at 590 nm measured on a microplate reader.

## Results and discussion

### The simulation model

**Fig. 1:**
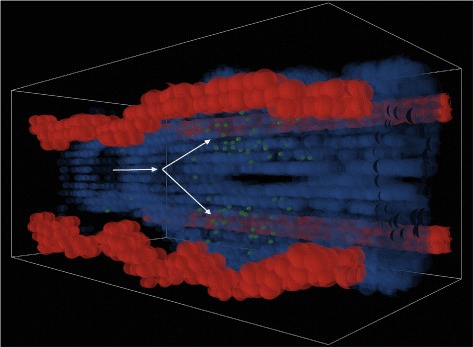
*Aspergillus fumigatus* spores in the airway. *A. fumigatus* spores (represented as *green spheres*) drift from one end of the airway to the other. The airway is lined with epithelial cells (*semi-transparent blue*); arrows indicate the direction of movement and the point at which the airway branches. Four adjacent capillaries (shown in *red*) run the length of the tissue segment

**Fig. 2:**
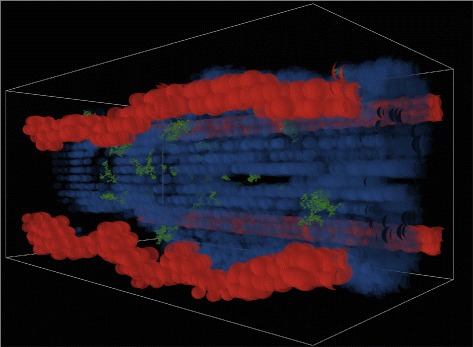
Clusters of germinated hyphae. Once spores have clung to the epithelial cell wall, they germinate and hyphal clusters (represented as clusters of *green cones*) grow through the epithelial wall and into the interstitial space

**Fig. 3:**
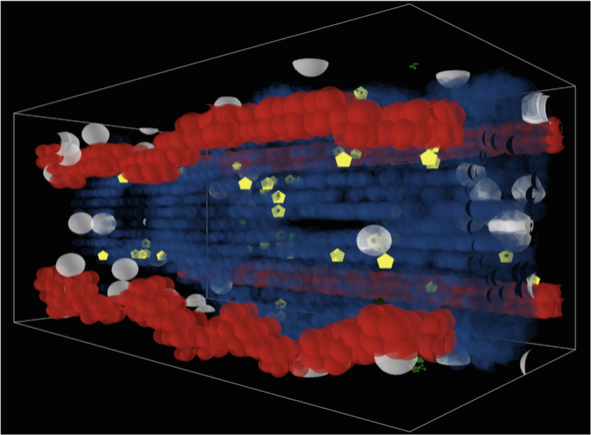
Immune response to fungal infection. Macrophages (*gray*) and neutrophils (*yellow*) are recruited to the site of infection via a chemotaxis gradient

The agent-based model consists of a three-dimensional simulation of a 400 *μm*×200 *μm*×200 *μm* section of lung tissue consisting of an alveolar duct, four adjacent capillaries, and surrounding lung parenchyma. The model was created in NetLogo [56], a popular platform for agent-based simulations. Figures 1, 2 and 3 provide still-frame snapshots from a three-dimensional dynamic simulation. *A. fumigatus* conidia enter at one end of the alveolar duct. As the simulation progresses, the conidia drift through the airway (see Fig. 1). The ABM simulates the epithelial clearance system via a parameter dictating the probability of conidia lodging in the epithelium. Left undisturbed, conidial spores enter a swelling phase prior to germination; subsequently, hyphal clusters begin to grow into the adjacent lung interstitium (see Fig. 2). In vivo, antimicrobial compounds in the airway surface fluid act as the first line of defense against conidia [57]; occasionally, conidia that are not cleared in this way can invade the interior of epithelial cells [58]; thus, the simulation also allows epithelial cells to internalize, damage, and kill conidia.

In the simulation model, epithelial cells act as the second line of defense against *A. fumigatus*. Epithelial cells recognize the presence of conidia and release inflammatory cytokines into the interstitial space in order to initiate an immune response. There are two immune cell types in the ABM: recruited monocytes/macrophages and neutrophils (see Fig. 3). We represent epithelial cells as releasing two types of chemotactic factors, for neutrophils and monocytes/macrophages, respectively. These chemotactic factors were tracked separately based on the literature [42, 59], and represent the aggregation of cytokines to which the respective immune cell types respond. The levels of these chemotactic factors are determined by the level of fungal burden, as measured by the number of conidia and hyphae. The chemotactic factors diffuse through the interstitial tissue, eventually reaching local capillaries. Once the local concentration for a particular immune cell rises above a threshold, they initiate recruitment of leukocytes adherent to the capillary vascular endothelium.

Recruited immune cells enter the interstitial space via the bloodstream. There, chemotaxis is simulated as the immune cells follow the gradient of the concentration of the chemotactic factor, a movement mechanism established in the literature [27, 28, 60]. At the source of the gradient, the immune cells may encounter fungal cells, whereupon such cells are attacked. Macrophages may internalize several fungal cells. Once internalized by a macrophage, cells are prohibited from escape and from germination. Over time, internalized fungal cells are damaged and ultimately destroyed. Damage inflicted by macrophages is limited by the number of internalized fungal cells, while neutrophil damage is limited by the number of available granules.

In the absence of sufficient chemotactic factors, immune cells move randomly throughout the tissue. Since the lifetime of neutrophils is between 24 and 48 h [61], neutrophils are represented as dying after this period. Macrophages leave the represented cross section when no conidial spores remain.

**Fig. 4:**
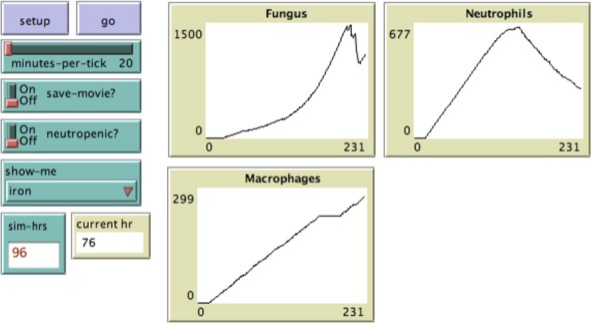
The interface for the simulation model

A key focus of the model is fungal acquisition of iron: the immune response induces hemorrhage, causing the tissue iron level to increase. The fungus is able to acquire iron both from the store of free iron and via a siderophore system. We model the level of available iron by having iron diffuse throughout the tissue. Once a certain level of iron is acquired by a fungal cell (determined by a parameter in the model), a new hyphal cell grows. Upon encountering hyphal cells, neutrophils sequester all of the iron in their immediate environment, preventing the fungus from acquiring it. If the immune system is not able to prevent the fungus from acquiring iron and consequently growing, invasive aspergillosis develops. The simulation continues in this manner until a pre-determined amount of time has passed. See Fig. 4 for a snapshot of the model interface, showing plots for various cell counts and model settings.

This description of the modeling of the immune response to *A. fumigatus* serves as an overview of the ABM and how the agents function. Many of the agent behaviors and parameter values are established in the literature; for a formal description of the model see Additional files 1 and 2. The ABM uses a combination of established behaviors, parameter settings, and the parameter analysis described below, building on previous work. Next we introduce results from parameter sensitivity analysis, which indicates the robustness of the model and offers insight into mechanisms of invasive aspergillosis in the lung.

### Parameter sensitivity

**Fig. 5:**
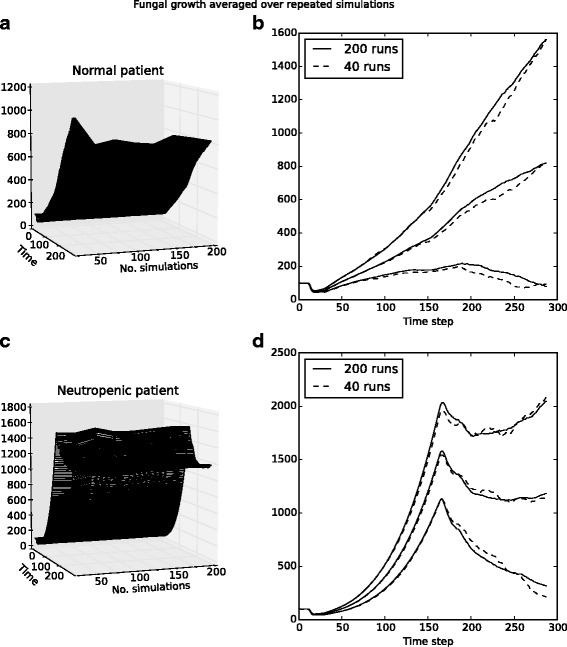
Determining suitable runs for reliable data over 288 time steps (96 h). Panels **a** and **c** indicate that fungal growth remains consistent at 200 simulations, and panels **b** and **d** indicate that in both immunocompetent and neutropenic simulations, mean and standard deviation over 40 simulations are very similar to the same data over 200 simulations (the middle curve is the mean; upper and lower curves show +/−1 standard deviation)

We have chosen four quantities from the ABM to compare with laboratory time-course data: fungal burden, iron level throughout the tissue space, macrophage cell counts, and neutrophil cell counts. We simulate normal immune system conditions as well as neutropenia. Neutropenia is induced in the simulation model in a manner similar to the laboratory setting: presence of neutrophils in tissue is ablated for the first 48 h, and then resumes to normal levels over the remaining duration of the simulation. While certain aspects of the ABM simulation are fixed (e.g. number of initial conidia, lifespan of monocytes/neutrophils, killing rates of epithelial cells), there remains variation from run to run as many processes are pseudo-random. In order to obtain reliable results from simulation, we examined the quantities of interest over many runs, and used the average and the standard deviation to determine the typical behavior of the ABM with respect to these quantities. Data for all quantities except fungal growth were very reliable when averaged over 20 simulations (in both neutropenic and healthy patients); for fungal growth it was necessary to run 40 simulations in order to obtain reliable data. Figure 5 provides a summary of this data for both healthy and neutropenic patients. In this figure we see that the mean value from 40 simulations is very similar to the mean taken over 200 simulations, and the data indicate that there is no significant change expected beyond 200 runs. Thus all results were generated by using the mean over 40 runs at each setting, with error bars representing one standard deviation.

While many state variable values have been determined from the literature, there are nine parameters whose values had to be determined empirically. Parameter descriptions and baseline values are provided in Table 1. With one exception, each parameter was set to 10 *%*, 50 *%*, and 200 *%* of its baseline value (one at a time) in order to examine the robustness of model results with respect to the parameter value; simulations were run under both healthy and neutropenic conditions. Due to higher sensitivity, one parameter – the radius within which macrophages and neutrophils can detect fungal presence – was set to 12 *μm*, 15 *μm*, and 18 *μm*. Full results for both normal and neutropenic conditions are provided in Additional file 3; an overview of the results follows.

**Table 1:** Parameters for sensitivity analysis and validation. The effects of these parameters were examined by setting each to 10 *%*, 50 *%*, and 200 *%* of their baseline values, which are indicated here. The final column provides values used for validation with laboratory data

Description	Name	Baseline	Validation
Probability of conidia lodging in epithelium	*p*_*lodge*_	0.05	0.0223
Macrophage / neutrophil fungal detection radius	*d* *e* *t*_*r* *a* *d* *i* *u* *s*	15 *μ* *m*	11.67*μ* *m*
Diffusion rate for iron and cytokines	*d* *i* *f* *f* *u* *s* *i* *o* *n*_*r* *a* *t* *e*	0.5	0.397
Cytokine production factor	*c* *y* *t* *o*_*r* *a* *t* *e*	100	100
Maximum iron acquirable by fungus	*i* *r* *o* *n*_*max*_(*f*)	2.5	3.633
Proportion of iron absorbed by fungus	*i* *r* *o* *n*_*a* *b* *s*(*f*)	0.5	0.76
Iron needed for fungal growth	*i* *r* *o* *n*_*min*_(*f*)	0.25	0.9096
Cytokine absorption rate by macrophages / neutrophils	*c* *y* *t* *o*_*a* *b* *s* *o* *r* *b*	0.05	0.05
Cytokine recruitment threshold for macrophages / neutrophils	*recr*	5	5

Three of the nine parameters had no qualitative effect (and little quantitative effect) on model dynamics. The first of these, the cytokine production factor, is a multiplier that determines the unitless amount of macrophage- and neutrophil-specific cytokines that is produced by epithelial cells in response to fungal presence. The lack of effect is likely a result of the fact that macrophages and neutrophils follow cytokine gradients to determine movement; since chemotaxis is simulated based on relative levels of nearby cytokines, the actual multiplier is not critical. The second of these parameters is the proportion of nearby cytokines that are taken up by receptors on macrophages and neutrophils. This lack of a significant effect can be explained in a similar way: cytokine uptake does not have a sufficiently large effect on the relative nearby cytokine concentration to alter cell movement. The final parameter to have little effect is referred to as the recruitment threshold; this parameter determines both the minimal amount of cytokine that must be present in order for macrophages and neutrophils to move according to chemotaxis (if all nearby areas are below the threshold they move randomly) and the possibility for recruited macrophages and neutrophils to appear in the blood (new immune cells appear only if their respective cytokine levels are above the threshold). This parameter serves two roles. If there is no threshold for initiating chemotaxis, immune cells quickly become fixed in a single location and do not move at all. Additionally, there must be a minimal cytokine level in the bloodstream in order to initiate immune cell recruitment. This parameter’s lack of effect can possibly be due to it being relatively small with respect to the cytokine production factor: as long as cytokines are being produced, the minimal threshold requirement is more or less arbitrary, and serves only to keep the immune cells moving, and to ensure that they are not being continuously recruited without reference to what is happening in the system. Thus, in all three cases it is not surprising that the parameter scaling had little effect on model dynamics.

**Fig. 6:**
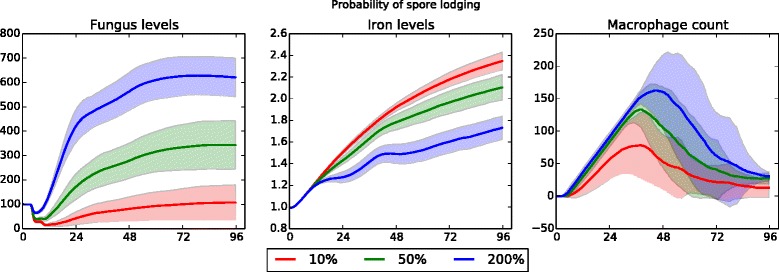
Data sensitivity to probability of conidia lodging in epithelium (healthy patient). Fungus, iron, and macrophage levels show quantitative differences, but the qualitative similarity indicates the simulation model is robust with respect to the probability of conidial spores lodging in the epithelium

Four more parameters resulted in unsurprising quantitative (but not qualitative) differences. The first of these is the probability of conidial spores lodging in the epithelium – this can be thought of as the strength of the cilia in sweeping away the fungus. Figure 6 shows the effect of this parameter in an immunocompetent host simulation. As the probability of lodging increases, fungal growth increases. In turn, iron levels decrease as the fungus takes up more of the available iron. Higher fungus levels cause an increase in cytokine production, which leads to an increase in recruited macrophages. The second parameter that scales predictably is the proportion of available iron that is absorbed by fungal cells. Given a fixed amount of iron needed for growth, the lower this proportion is the less resultant fungal growth; at the same time, because there is less fungal growth there is an increase in systemic iron levels and a slightly lower macrophage count. However, when taking into account the variance of the data, the only significant difference is in fungal growth. The third parameter to scale quantitatively is the iron needed in order for fungus to grow. As this level is decreased, fungal growth increases; iron levels and macrophage counts remain approximately the same.

**Fig. 7:**
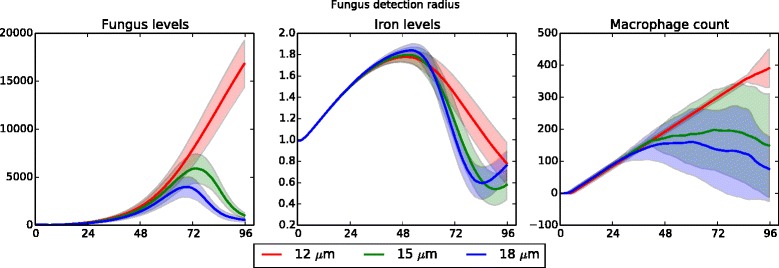
Data sensitivity to detection radius of immune cells (neutropenic patient). Fungal growth and iron levels are highly sensitive with respect to the detection radius; the macrophage cell count is sensitive to a lesser degree

The fourth parameter in this category is the detection radius, which determines how close an immune cell needs to be in order to detect fungal presence. As noted above, model dynamics were more sensitive to this parameter than to any of the others. These results are highlighted in Fig. 7, which summarizes results in a neutropenic simulation. When the detection radius was set to be 12 *μm*, fungal growth is rapid and hardly affected at all by the presence of immune cells. However, by changing the detection radius to just 15 *μm* the dynamics were drastically altered: practically all of the fungus is removed by the immune cells. This is most likely an artifact of the way Netlogo handles distance – at 12 *μm*, only the grid location of the immune cell is considered, whereas at 15 *μm* each of the 26 neighbors (in three dimensions) are considered. Thus, in light of this, these results are not so surprising. From a detection radius of 15 *μm* to 18 *μm*, the results scale quantitatively in the expected manner: a higher detection radius results in less fungal growth and fewer macrophages, though iron levels remain fairly consistent.

**Fig. 8:**
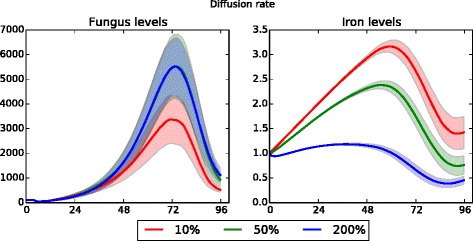
Data sensitivity to iron and cytokine diffusion rate (neutropenic patient). As the diffusion rate is lowered, fungal growth decreases and available iron increases

The remaining two parameters subjected to sensitivity analysis are open to different interpretations, and offer interesting insights into the dynamics of the system. The first of these is the diffusion rate of iron and cytokines throughout the tissue. As the diffusion rate is lowered, the fungal growth rate is decreased, resulting in higher total iron levels in the system (as less iron is being consumed by the fungus); see Fig. 8 for results from neutropenic simulations. The diffusion rate does not have any significant effect on the macrophage count, as their presence is governed almost entirely by cytokine levels. Interestingly, at the highest diffusion rate (1.0), the total systemic iron is lower than when the diffusion rate is set to 0.25, but the fungal growth in each of these cases is the same. This effect is observed in both neutropenic and immunocompetent simulations. This may be because the macrophages are fighting off the fungus and thus controlling the fungal growth rate; in any event the result suggests that lowering iron diffusion might be an effective way to increase systemic iron levels without increasing fungal growth.

**Fig. 9:**
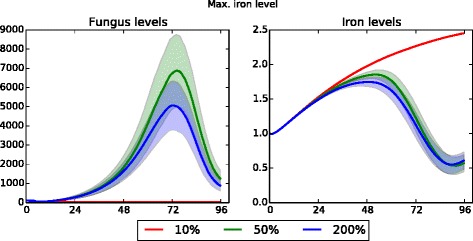
Data sensitivity to maximum iron acquirable by fungal cells (neutropenic patient). Lower maximum stored iron inhibits fungal growth and results in higher systemic iron levels and a decrease in the macrophage cell count

The last parameter subjected to sensitivity analysis is the maximum amount of iron that fungal cells can store; the data from a neutropenic simulation is presented in Fig. 9. As the amount of iron coming into the system is fixed (at a value of 1 unit per hour), the maximum iron level was set to 0.25, 1.25, and 5.0 (representing 10 *%*, 50 *%*, and 200 *%* of the fixed baseline value of 2.5 used in analysis of the other parameters). It is important to note that the amount of iron required for growth in each of these cases was fixed at the baseline value of 0.25. Thus when the maximum iron level is set to 0.25, the fungus is unable to grow; this allows systemic iron levels to rise, and since there is no fungus, fewer macrophages are recruited. These results suggest that even in neutropenic cases, fungal growth can be inhibited if the iron acquisition system is disabled.

Additional file 3 provides full data from the parameter analysis for both immunocompetent and neutropenic simulations; the results shown here summarize the most relevant findings. Data from neutropenic simulations are presented because in general, the effects are more pronounced in this setting. However, the same results hold for immunocompetent host simulations. It is worth noting that in all cases, neutrophil cell counts are not included as they are not significantly affected by different parameter values. In immunocompetent hosts, only the diffusion rate and the cytokine production factor affect neutrophil recruitment, and very slight effects are observed. In neutropenic simulations, no difference is observed. Due to the way in which neutropenia is simulated (as described above), it is not surprising that the parameters have little effect on neutrophil cell counts.

The parameter sensitivity analysis performed here is rudimentary and largely serves to examine the robustness of the system. Given that data are coming from stochastic simulations, a more traditional sensitivity analysis is computationally infeasible. Nevertheless, this analysis brings forth several interesting hypotheses. The dependence on iron for fungal growth cannot be considered a prediction of the model, as it is built into the code that dictates fungal behavior. However, the effect of iron and cytokine diffusion and iron storage on fungal growth indicate a possible means by which invasive aspergillosis can be avoided, even in the neutropenic case. It would be possible to investigate these predictions in future laboratory work. These results suggest that the model is robust with respect to the nine unvalidated parameters, indicating the strength and viability of using the simulation model to investigate invasive aspergillosis in the lung.

### Validation

In vivo experiments were carried out for immunocompetent mice and those in whom neutropenia was induced, in order to study the effectiveness of the immune response under both conditions. Accordingly, simulations of the *in silico* model include data for both immunocompetent and neutropenic mice. In order to mimic in vivo conditions in neutropenic simulations, neutrophil levels are depleted gradually over the course of the first 24 h, n entirely for the following 48 h, and returned to full production over the course of the subsequent 24 h.

**Fig. 10:**
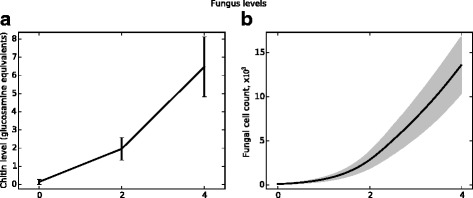
Fungal growth from experimentation (panel (**a**)) and ABM data from simulations of neutropenia (panel (**b**)). Data shown in panel **a** represent mean and SEM of *n*=4-6 mice per time point

**Fig. 11:**
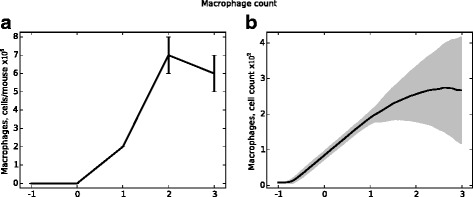
Recruited macrophage counts from in vivo experiments (panel (**a**)) and from the ABM (panel (**b**)). Data shown in panel **a** represent mean and SEM of *n*=4-9 mice per time point

**Fig. 12:**
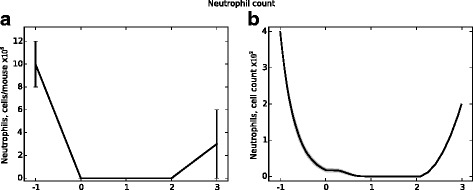
Neutrophil counts from in vivo experiments (panel (**a**)) and from the ABM (panel (**b**)). Data shown in panel **a** represent mean and SEM of *n*=4-9 mice per time point

**Fig. 13:**
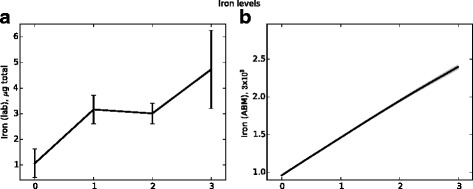
Iron levels from in vivo experiments (panel (**a**)) and from the ABM (panel (**b**)). Data shown in panel **a** represent mean and SEM of *n*=4-6 mice per time point

Chitin is a carbohydrate component of the hyphal wall that is absent from conidia and mammalian tissues. Chitin levels measured in vivo therefore indicate hyphal presence; as such, chitin levels are used as an indicator of total fungal burden. Figure 10 presents in vivo chitin levels and fungal cell counts from the agent-based model. Figures 11 and 12 present similar data (from the same simulations) for macrophage and neutrophil cell counts, respectively. In the data presented, a neutrophil-depleting antibody is given one day prior to the introduction of conidia; hence the time scales begin at day −1. Fungal spores are introduced in the ABM beginning at day 0. Figure 13 shows the qualitative similarity between experimental iron data and iron levels in the simulation. Parameter values for these figures were determined by a heuristic search method implemented directly in NetLogo; the values used to produce these figures are given in Table 1.

## Conclusion

The immune response to fungal and other pathogens in the lung is multi-faceted and multi-scale. Thus, it lends itself well to a systems biology approach based on mathematical modeling. Our goal underlying the work presented here is to lay the groundwork for a multi-scale dynamic mathematical model of the innate immune response to *A. fumigatus* in the lung, focused on the “battle for iron” between host and pathogen. Iron acquisition has been shown to be an important virulence factor for the fungus, and several mechanisms of the immune response are designed to adversely affect fungal iron uptake. The model we constructed covers the tissue scale and incorporates recognition of the fungus by epithelial cells, production of chemotactic gradients and recruitment of macrophages and neutrophils, as well as changes in tissue-level iron concentrations. However, this is accomplished through rules that do not do full justice to the intracellular processes triggered by pathogen recognition. In a future multi-scale version of the model, these processes will be controlled through intracellular signaling and iron regulation mechanisms.

In any computational model, required approximations inevitably give rise to limitations. In the model presented here, one of these is the absence of volume exclusion; that is, that multiple cells may aggregate in a manner that is not physically feasible. While not indicated by preliminary simulation results, it is possible that cell crowding has an effect on the results of the model. The decision to not incorporate volume exclusion was motivated by the relatively low quantity of immune cells and the computational complexity required to model using volume exclusion. Incorporation of an increased variety of immune cell types would also increase the potential effectiveness of the model, as would incorporation of multiple cytokines rather than the cell-specific aggregates implemented here. A better understanding of how immune cells sequester iron, particularly in a quantitative sense, would also allow the model to be fine-tuned in a more realistic manner. The use of unit-less proportions to determine iron levels (and diffusion) act as another limitation, and one that could be addressed by biological experimentation. All of these limitations may be investigated in future versions of the model.

Parameter sensitivity analysis was conducted to ensure that the model is robust, and data was validated by fitting model parameters to data from a neutropenic mouse model. The computational model can capture the fungal burden qualitatively over time, using chitin levels as proxy, and the recruitment of macrophages, while reproducing leukocyte counts. Thus, this study can be considered as proof of the concept that a computational model of this kind can serve as a tool to study invasive aspergillosis. Some parameter values were determined from literature while others were determined experimentally; in this way, parameter values arose quite naturally from studies focusing on individual cells (for full model details see Additional files 1 and 2). In order to increase the realism of the model, we plan to add other cells (such as dendritic cells and natural killer cells); we then plan to endow all cells with intracellular networks that capture the signaling and metabolic processes involved. Such a multi-scale model can then be used to test hypotheses *in silico* and to explore therapeutic interventions.

## Availability of data and materials

The data supporting the results of this article are included within the article.

## Consent to publish

Not applicable.

## Ethics

Animal studies were carried out in accordance with the Guide for the Care and Use of Laboratory Animals of the National Institutes of Health and were approved by the Animal Studies Committee at University of Virginia School of Medicine.

## Additional files

Additional file 1 Overview, Design Concepts, and Details (ODD) protocol for the agent-based model. A complete description of the agent-based model, including process ordering, state variable values, and algorithm pseudocode (in.pdf format). (PDF 145 kb)

Additional file 2 State variables for the agent-based model. Descriptions and values for all state variable values, organized by cell type (with global variables indicated as such). *N*(*μ*,*σ*)indicates values taken from a normal distribution with mean *μ* and standard deviation *σ* (in.pdf format). (PDF 985 kb)

Additional file 3 Experimental design and results of parameter sensitivity analysis. Parameters not validated by literature are altered to 10 *%*, 50 *%*, and 200 *%* of baseline values in order to determine the effect of each (one at a time). Relevant model dynamics are presented under both normal and neutropenic conditions (in.pdf format). (PDF 186 kb)

## Abbreviations

ABM: agent-based model
