# Supplementary material for: A computational model of invasive aspergillosis in the lung and the role of iron
Source: BMC Syst Biol. 2016 Apr 21;10:34. doi: 10.1186/s12918-016-0275-2 (PMC4839115; doi:10.1186/s12918-016-0275-2)
Supplement: Additional file 1 — Overview, Design Concepts, and Details (ODD) protocol for the agent-based model. A complete description of the agent-based model, including process ordering, state variable values, and algorithm pseudocode (in.pdf format). (PDF 145 kb) [file 12918_2016_275_MOESM1_ESM.pdf]

Overview, Design Concepts, and Details protocol for an agent-based model of *A. fumigatus* in the lung

The Overview, Design Concepts, and Details (ODD) protocol for describing agent-based models was first introduced by Grimm in 2006 [1] and subsequently updated in 2010 [2]. The purpose of the protocol is to serve as a standard template for ABM description, including detail sufficient enough that models can be replicated independently. This document provides the ODD protocol for the model introduced in the body of the paper. The model is described in full detail, including citations to how certain state variable values were chosen. A master table of state variables is provided in Table S2. Pseudocode indicating precise process scheduling is provided.

A.1 Purpose

The purpose of this model is to simulate the human immune response to *Aspergillus fumigatus* in the lung. Specifically, the purpose is to investigate the following questions:

- What is the role of iron in both immune response and fungal growth?
- How does invasive aspergillosis develop in immunocompromised patients?

A.2 Entities, state variables, and scales

In this section, global state variables, model parameters, and spatial and temporal scales are described first, followed by behavioral descriptions for grid cells and entities. A table of state variable values is provided in Table S2. Initialization values are given in Section A.5.

Global state variables, model parameters, and scales. This model is a simulation of the effects of an inoculation of *A. fumigatus* spores on a cross-section of lung tissue. As such, the inoculum refers to that given to the entire patient; only a fraction of those spores appear in any cross-section. The spatial scale was chosen for computational and visual considerations. As patient fate is typically determined in the first four days after inoculation [3], 96 hours was chosen as the virtual duration of a simulation.

Grid cells. The **airway** is a tube of grid cells which branches about one-third of the way across the horizontal span of the model. In total, approximately 16% of the grid cells make up the airway. Airway grid cells have no properties other than the number of spores at that location. Non-specific **interstitial** tissue cells comprise approximately 80% of the grid cells. The state variables for these cells are iron, macrophage-specific cytokines, and neutrophil-specific cytokines. Four blood vessels run the length of the model, made up of **blood cells** occupying one grid cell each; they comprise approximately 4% of the grid cells. These cells serve as the recruitment sites for macrophages and neutrophils as well as the source of iron for the interstitial space. Iron is introduced in the blood cells and diffuses into surrounding tissue. Cytokine levels are first affected by the epithelial cells and diffuse from the epithelium throughout the interstitial and blood cells. These cytokines serve as aggregations of macrophage- and neutrophil-specific cytokines, such as TNF-α, IL-8, IFN$_\gamma$, and others.

Fungal spores. *A. fumigatus* spores drift through the airway, and if a spore meets the epithelial cell wall it lodges there with a fixed probability. This probability is small, as ciliary beating serves as the primary mechanism for elimination of the fungus [4]. Once lodged, a spore cannot be swept away. Spores that are swept away are placed on the nearest airway grid cell and continue to drift. Once a spore has traversed the entire airway, it is removed from the simulation.

Approximately 30% of spores are internalized by an epithelial cell [5]. Since approximately 3% of internalized spores survive and 34% of these germinate [6], internalized spores germinate with approximate probability 0.01. Internalized or not, all lodged spores enter a resting stage. Upon completion of the resting stage, all non-internalized spores become swollen. After remaining in the swollen stage for some time, spores germinate. Since the resting stage lasts roughly 2 hours and germination occurs between 6 − 8 hours [7], the swelling process lasts approximately 5 hours. A germinated spore spawns a fungal hyphal cell, which is connected to the spore and grows into neighboring tissue in a random direction. Since behavior of hyphae are different from that of fungal spores, these agents are described separately.

Epithelial cells. Epithelial cells form the boundary between the airway and the interstitial tissue. Each time step, these cells first determine the number of fungal spores and hyphae within their detection radius. These counts indicate the amount of macrophage-specific and neutrophil-specific cytokines to produce, respectively. While the cells produce the cytokine levels based on fungal presence, these levels are in fact grid cell state variables, not attributes of the epithelial cells. After adjusting local cytokine levels accordingly, each epithelial cell damages any internalized spores. This continues until either the spore germinates and kills the epithelial cell, or the spore dies. It is observed in [8] that only 3% of conidia survive at least 36 hours – hence 30 hours is chosen as the approximate time it takes an epithelial cell to kill an internalized conidium.

Macrophages. Macrophages are recruited to the site of infection via the bloodstream. Each time step, if there is at least one blood cell whose macrophage-specific cytokine level is above the macrophage recruitment threshold, there is a possibility of a macrophage spawning at exactly one such location. All macrophages absorb some of the macrophage cytokines on their current location, simulating uptake by receptors. Macrophages have a detection radius for conidial spores and hyphae; if a macrophage has fewer than two internalized spores, it will internalize a spore within this radius. Internalization prevents spores from growing hyphae. The internalization process takes approximately two hours [9]; since engulfed conidia are assumed unable to escape, this is used as the time it takes for a macrophage to kill an internalized spore. Macrophages do not phagocytose hyphae, but they are known to produce IL-8 to aid in neutrophil recruitment [10, 11] – this is simulated by having macrophages increase the neutrophil-specific cytokine level at their current location based on the amount of nearby hyphae.

Fungal hyphae. Hyphae grow out of germinated fungal spores. They are attached to the spores and thus do not move. Once a hyphal cell appears, it spends time in a growth stage, during which no new hyphae can grow out of it. During the growth phase, if the hyphae has not been internalized, it attempts to absorb iron from surrounding tissue. Once the cell obtains enough iron, a new hyphal cell (or two new cells, if branching occurs) grows from the parent cell, with the new cells inheriting iron from the parent. Once it has spawned a new cell, a parent cell is no longer eligible for spawning future cells, though it does continue to take up iron from the environment. Many hyphae state variable values are unitless, abstract measures (e.g., iron levels), while others are chosen empirically.

Neutrophils. Neutrophils are recruited from the bloodstream in much the same way as macrophages – if there is at least one blood cell with neutrophil-specific cytokines above a certain threshold, then there is the possibility of a neutrophil spawning at exactly one such location. To indicate absorption of cytokines by receptors, every neutrophil reduces the neutrophil-specific cytokine level at its current location. As long as there are grid cells nearby with cytokine levels above the recruitment threshold, neutrophils move towards the grid cell with highest cytokine level – this simulates the chemotactic movement of recruited neutrophils [12]. Otherwise, they move to the center of a randomly chosen neighboring non-airway grid cell. Neutrophils raise cytokine levels in order to boost recruitment of additional neutrophils by an amount equal to the number of hyphae within their detection radius. Neutrophils are initialized with a fixed number of granules, which are deposited every time step in order to degrade and kill hyphae. Various studies indicate that the approximate killing time of hyphae by neutrophils is 2 hours [13, 14]. All hyphae within the neutrophil detection radius are damaged as long as the neutrophil has not run out of granules; during this process iron is removed from the neutrophil's location in order to simulate sequestration. Additionally, neutrophils do not move while they are degrading nearby hyphae. Recruited neutrophils have a lifespan of 1 – 2 days [15]; thus in the model, the average lifespan is set at 36 hours.

A.3 Process overview and scheduling

In the simulation, time is discrete. Entity routines are executed serially – that is, one entity executes every command in the routine, then the next entity does the same, and so on. This means that entity state variables are updated asynchronously; pertinent state variables are updated as each command is executed. The order in which entities perform the routine is randomly chosen at each time step. The pseudocode in Algorithm S3.1 describes the global process overview; sub-processes are provided in Section A.7. State variables referred to in the algorithms are described in Table S2.

Algorithm S3.1 Pseudocode for the entire model process.

```
setup map and constants
while current_sim_time < total_sim_time do
    conidia routine:
        if health_f ≤ 0 then die end if
        if mobile then
            if at edge of model then die else conidia move end if
            check for contact and lodging with epithelium
            if lodged then
                check for internalization
                begin growth countdown
            end if
        end if
        growth countdown
        if growth time reached then
            switch stage of spore do
                case swollen: germinate
                case resting and not internalized: become swollen
                case internalized: swell with probability p_swell
            if swollen then begin swelling countdown end if
        end if
        iron uptake
        grow hyphae
    end routine
    iron diffusion
    epithelial cytokine update
    epithelial damage internal spores
    cytokine evaporation and diffusion
    check for new macrophages
    macrophage absorb cytokines
    if any free spores near macrophages then macrophages internalize spore end if
    macrophage produce neutrophil cytokine
    macrophage move
    macrophage damage conidia
    check for new neutrophils
    neutrophil absorb cytokines
    neutrophil produce neutrophil cytokine
    neutrophil move
    neutrophil damage hyphae
    increase age according to time scale
    if age limit reached then die end if
    increase time by time_step
end while
```

A.4 Design concepts

Basic principles. The biological mechanics of cell-cell interactions are the basic principles underlying the design of this model. These are described in literature and derived from experimentation, both *in vivo* and *in vitro*. Some behavioral processes, such as phagocytosis, occur on a local scale between two cells, while others, such as recruitment of macrophages and neutrophils, are tissue-level processes. The model aims to tie together what is known about individual cell behavior to create an *in silico* model of a larger-scale process.

Emergence. The ability of the fungus to survive under various conditions is an emergent property of individual fungal cells competing for iron. At the same time, the tendency for survival of infection is an emergent property of the entire immune response.

Adaptation. Macrophages and neutrophils both generally travel in the direction of highest cytokine concentration; thus, they adapt their movement in response to developing infection.

Objectives. The objective of immune cells is to remove all fungal cells, which is measured simply as a count of those cells. Thus whether a patient lives or dies depends on the amount of fungus present in the system.

Learning. Agents do not change adaptive traits over time in this simulation.

Prediction. Agent prediction is similar to adaptation in this case: macrophages and neutrophils inherently predict that following the path of highest cytokine concentration will lead to the area where immune response is most needed.

Sensing. Fungal spores sense the type of grid cell they are near, be it airway or interstitial space. In addition, both spores and hyphae sense the amount of iron present at their current location, and use this information to 'decide' if they will grow. Epithelial cells, macrophages, and neutrophils can all sense nearby spores and hyphae as well as the cytokine levels of all neighboring cells. All sensing is local.

Interaction. While section A.2 provides a fairly comprehensive overview of entity interactions, it is perhaps helpful to mention several interactions which do **not** occur. Specifically, macrophages and neutrophils do not directly interact with other immune cells, either of their type or another. For example, the presence of many macrophages at one location has no bearing on the likelihood of another macrophage targeting that location. Fungal hyphae interact with other hyphae as long as they are connected − in particular, iron uptake is shared among all hyphae at a given location, and when new hyphae are spawned, they inherit iron from the parent cell.

Stochasticity. Agent movement, when not dictated by concerns such as cytokine levels, is stochastic: agents face in the direction of a randomly chosen legal space (i.e., non-airway) and move in that direction. Hyphae grow in random directions, leading to clumps of fungus rather than long strands; hyphal branching is stochastic as well. In general, stochasticity is employed as a substitute for unknown mechanisms of the biological entities being represented.

Collectives. Fungal hyphae form a collective of sorts, as they maintain information about every other hyphae they are connected to. Iron stores are shared among local neighbors, which has an indirect effect on the collective group. These groups arise naturally and from the local rules of individual entities; they are not entities themselves and have no distinct state variables.

Observation. Many data can be collected from the model for analysis and understanding. These include fungal cell counts, iron levels, cytokine levels, macrophage and neutrophil counts, and collateral tissue damage. Any or all of these may be collected during each simulation, depending on the needs and interests of the researcher. The data are collated in a universal spreadsheet format for ease of statistical interpretation and analysis.

A.5 Initialization

Many initialization values are given in Table S2; as such, they are not repeated here. In addition to these values, the following information may be necessary to reproduce results: blood and interstitial tissue cells begin with iron at 20; all grid cells begin with cytokine levels at 0. The initial inoculum is placed at one end of the airway. There are no macrophages or neutrophils until recruitment occurs via cytokine triggering. Macrophages and neutrophils are created at the blood cell where recruitment occurs. Given that the model is meant to function as an *in silico* laboratory, it is not meant to be entirely robust with respect to initial conditions. Indeed, a key feature of the model is the ability to investigate the effect of initial conditions on outcome.

A.6 Input data

The model requires a map file (in .txt format) in order to set up grid cell types and locations. The map file is a three-dimensional matrix. Grid cell layout is determined aesthetically to serve the visual representation of model dynamics. No other input data is used.

A.7 Submodels

All submodels are presented here as pseudocode. The notation $rand(a, b)$ indicates a uniformly distributed random number chosen from interval (a, b).

Algorithm S3.2 ***conidia move***

face random direction down airway
move forward $speed_f$
if at edge of map **then** die **end if** ▷ simulates inoculum passing through airway

Algorithm S3.3 ***iron uptake***

for non-airway grid cell here with $iron_p > cell\_iron_{min}$ **do**
    for all non-captive hyphal or hyphal-tip fungus here with $iron_f < iron_{max}(f)$ **do**
        $iron_f = iron_f + (iron\_abs(f) \cdot iron_p/\text{no. fungus here})$ ▷ iron is split among all eligible fungus
        $iron_f = \min\{iron_f, iron_{max}(f)\}$ ▷ iron capped at $iron_{max}(f)$
    end for
    $iron_p = (1 - iron\_abs(f)) \cdot iron_p$ ▷ grid cell iron reduced
end for

Algorithm S3.4 ***grow hyphae***

for all germinated non-captive spores which haven't spawned hyphae **do**
    spawn hyphal cell
    hyphal cell routine:
        move forward *spacing* in a random non-airway direction
        set stage to 'hyphal tip'
        set growth time to t_{grow} ▷ begin swelling phase
        $iron_f = iron_f$ of parent cell
    end routine
end for
for all hyphal tip cells with $iron_f > iron_{min}(f)$ and growth time reached **do**
    $num\_to\_spawn = 1$
    if $rand(0, 1) < p_{branch}$ **then** ▷ probability of branching
        $num\_to\_spawn = 2$
        $iron_f = iron_f/3$
    else
        otherwise $iron_f = iron_f/2$
    end if
    spawn num_to_spawn hyphal cells
    set stage to 'hyphal' ▷ only 'hyphal tip' cells can spawn
    each new hyphal cell performs **hyphal cell routine**
end for

Algorithm S3.5 ***iron diffusion***

for all blood grid cells **do**
    $iron_p = \min\{(iron_p + iph \cdot time\_step/60), iron_{max}(p)\}$ ▷ iron is capped at $iron_{max}(p)$
end for
for all non-airway grid cells **do**
    give each neighbor cell $(iron_{dif} \cdot iron_p/26)$ iron ▷ 26 neighbors in 3D
    airway cells: $iron_p = 0$ ▷ no iron in airway
end for

Algorithm S3.6 ***epithelial cytokine update***

for all epithelial cells **do**
    $spore\_count$ = swollen or germinated spores within s_{det}
    $hyphae\_count$ = hypal or hyphal-tip cells within h_{det}
    $cyto_m = cyto_m + cyto\_rate \cdot spore\_count$ ▷ increase cytokine level based on counts
    $cyto_n = cyto_n + cyto\_rate \cdot (hyphae\_count + spore\_count)$
end for

Algorithm S3.7 ***epithelial damage internal spores***

for all internalized spores **do**
    $health_f = health_f - (init\_health(f) \cdot time\_step/e_{kill})$
end for

Algorithm S3.8 ***cytokine evaporation and diffusion***

for all grid cells **do**
    $cyto_m = (1 - cyto\_evap\_m) \cdot cyto_m$ ▷ cytokine evaporation
    $cyto_n = (1 - cyto\_evap\_n) \cdot cyto_n$
    add $cyto_m/26$ to each neighboring cell ▷ $cyto_m$ refers to cytokine level of contributing cell
    add $cyto_n/26$ to each neighboring cell ▷ diffusion to 26 neighbors in 3D
end for
airway cells: $cyto_m, cyto_n = 0$ ▷ no cytokines in airway

Algorithm S3.9 ***check for new macrophages***

Repeat twice:
if any blood cells with $cyto_m \geq m_{recr}$ **then**
  let L be one such location, selected at random
  if $rand(0,1) < p_{recr}(m)$ **then** ▷ probability of recruitment
    spawn new macrophage at location L
  end if
end if

Algorithm S3.10 ***macrophage absorb cytokines***

$cyto_m = (1 - m_{abs}) \cdot cyto_m$

Algorithm S3.11 ***macrophage produce neutrophil cytokine***

let h be number of fungus in stage 'hyphal tip' or 'hyphal' within m_{det}
grid cell here: $cyto_n = cyto_n + m_n \cdot h$

Algorithm S3.12 ***macrophage move***

if any neighboring grid cells with $cyto_m \geq m_{recr}$ **then**
  move to neighbor with highest $cyto_m$ ▷ follow highest cytokine concentration
else
  move to random neighboring grid cell ▷ may wrap around map
end if

Algorithm S3.13 ***macrophage damage conidia***

for all internalized conidia **do** ▷ macrophages internalize spores only, not hyphae
  $health_f = health_f - (init\_health(f) \cdot time\_step/m_{kill})$ ▷ damage depends on time scale
  ensure location is same as macrophage
end for

Algorithm S3.14 ***check for new neutrophils***

let $num\_reps = 6$
if patient is neutropenic and $48 \leq current\_sim\_time \leq 96$ **then**
  set $num\_reps = (current\_sim\_time - 48)/8$ ▷ suppress neutrophil production between days 2 and 4
end if
Repeat num_reps:
if any blood cells with $cyto_n \geq n_{recr}$ **then**
  let L be one such location, selected at random
  spawn new neutrophil at location L
end if

Algorithm S3.15 ***neutrophil absorb cytokines***

$cyto_n = (1 - n_{abs}) \cdot cyto_n$

Algorithm S3.16 ***neutrophil produce neutrophil cytokine***

let h be number of non-internalized fungus in stage 'hyphal tip' or 'hyphal' within n_{det}
grid cell here: $cyto_n = cyto_n + N_n \cdot h$

Algorithm S3.17 ***neutrophil move***

if degranulating nearby hyphae **then**
  do not move
else if any neighboring grid cells with $cyto_n \geq n_{recr}$ **then**
  move to neighbor with highest $cyto_n$ ▷ follow highest cytokine concentration
else
  move to random neighboring grid cell ▷ may wrap around map
end if

Algorithm S3.18 ***neutrophil damage hyphae***

```
if any hyphae within n_det and gran > 0 then
    hyphae: health_f = health_f − (init_health(f) · time_step/n_kill)
    gran = gran − 1
    all grid cells within n_det: set iron_p = 0        ▷ neutrophils sequester iron
end if
```

References

[1] Grimm V, Berger U, Bastiansen F, Eliassen S, Ginot V, et al. (2006) A standard protocol for describing individual-based and agent-based models. Ecological Modelling 198: 115 - 126.

[2] Grimm V, Berger U, DeAngelis DL, Polhill JG, Giske J, et al. (2010) The ODD protocol: A review and first update. Ecological Modelling 221: 2760 - 2768.

[3] Hope WW, Petraitis V, Petraitiene R, Aghamolla T, Bacher J, et al. (2010) The initial 96 hours of invasive pulmonary aspergillosis: histopathology, comparative kinetics of galactomannan and $(1- > 3)$ β-d-glucan and consequences of delayed antifungal therapy. Antimicrob Agents Chemother 54: 4879–4886.

[4] Thompson AB, Robbins RA, Romberger DJ, Sisson JH, Spurzem JR, et al. (1995) Immunological functions of the pulmonary epithelium. Eur Respir J 8: 127–149.

[5] Wasylnka JA, Moore MM (2002) Uptake of Aspergillus fumigatus Conidia by phagocytic and nonphagocytic cells in vitro: quantitation using strains expressing green fluorescent protein. Infect Immun 70: 3156–3163.

[6] Wasylnka JA, Moore MM (2003) Aspergillus fumigatus conidia survive and germinate in acidic organelles of A549 epithelial cells. J Cell Sci 116: 1579–1587.

[7] Manavathu EK, Cutright J, Chandrasekar PH (1999) Comparative study of susceptibilities of germinated and ungerminated conidia of Aspergillus fumigatus to various antifungal agents. J Clin Microbiol 37: 858–861.

[8] Botterel F, Gross K, Ibrahim-Granet O, Khoufache K, Escabasse V, et al. (2008) Phagocytosis of Aspergillus fumigatus conidia by primary nasal epithelial cells in vitro. BMC Microbiol 8: 97.

[9] Philippe B, Ibrahim-Granet O, Prevost MC, Gougerot-Pocidalo MA, Sanchez Perez M, et al. (2003) Killing of Aspergillus fumigatus by alveolar macrophages is mediated by reactive oxidant intermediates. Infect Immun 71: 3034–3042.

[10] Kohidai L, Csaba G (1998) Chemotaxis and chemotactic selection induced with cytokines (IL-8, RANTES and TNF-alpha) in the unicellular Tetrahymena pyriformis. Cytokine 10: 481–486.

[11] Wang JE, Warris A, Ellingsen EA, Jorgensen PF, Flo TH, et al. (2001) Involvement of CD14 and toll-like receptors in activation of human monocytes by Aspergillus fumigatus hyphae. Infect Immun 69: 2402–2406.

[12] Dagenais TR, Keller NP (2009) Pathogenesis of Aspergillus fumigatus in Invasive Aspergillosis. Clin Microbiol Rev 22: 447–465.

[13] Diamond RD, Clark RA (1982) Damage to Aspergillus fumigatus and Rhizopus oryzae hyphae by oxidative and nonoxidative microbicidal products of human neutrophils in vitro. Infect Immun 38: 487–495.

[14] Rex JH, Bennett JE, Gallin JI, Malech HL, Melnick DA (1990) Normal and deficient neutrophils can cooperate to damage Aspergillus fumigatus hyphae. J Infect Dis 162: 523–528.

[15] Stevens A, Lowe JS, Young B (2002) Wheater's Basic Histopathology: A Color Atlas and Text (Wheater's Histology and Pathology). Churchill Livingstone, 4 edition.
