# Supplementary material for: A computational model of invasive aspergillosis in the lung and the role of iron
Source: BMC Syst Biol. 2016 Apr 21;10:34. doi: 10.1186/s12918-016-0275-2 (PMC4839115; doi:10.1186/s12918-016-0275-2)
Supplement: Additional file 2 — State variables for the agent-based model. Descriptions and values for all state variable values, organized by cell type (with global variables indicated as such). N(μ,σ)indicates values taken from a normal distribution with mean μ and standard deviation σ (in.pdf format). (PDF 985 kb) [file 12918_2016_275_MOESM2_ESM.pdf]

Table 1: **Model initialization variables.**

State variable type	Description	Name	Value	Units
global	world size	$world\_size$	$20 \times 40 \times 20$	grid cells
grid cell	grid cell length	$grid\_size$	10	μm
blood cell	iron per hour	iph	1	none
conidia	number of initial spores	$init\_inoc$	100	spores

Descriptions and values for model initialization values.

Table 2: **State variables with literature citations.**

State variable type	Description	Name	Value	Units	Reference
global	total simulation time	$total\_sim\_time$	96	hours	[2]
epithelial cell	time taken to kill internal spore	e_{kill}	30	hours	[11]
fungal hyphae	spacing of hyphal cells	$spacing$	$N(1.67, 0.16)$	$\mu m/hr$	[8]
fungal spore	drift speed	$speed_f$	$N(2.2, 0.2)$	$\mu m/min$	[6]
fungal spore	prob. of internalization	p_{int}	0.3	N/A	[10]
fungal spore	prob. of internal spore swelling	p_{swell}	0.01	N/A	[1]
fungal spore	duration of resting stage	t_{rest}	$N(120, 10)$	minutes	[4]
fungal spore	duration of swollen stage	$t_{swollen}$	$N(300, 30)$	minutes	[4]
fungal spore	duration of growth stage	t_{grow}	$N(105, 10)$	minutes	[4]
macrophage	time taken to kill internal spore	m_{kill}	$N(120, 10)$	minutes	[5]
macrophage	movement speed	$speed_m$	$N(6, 1)$	$\mu m/min$	[6]
neutrophil	time taken to kill hyphae	n_{kill}	$N(120, 30)$	minutes	[7]
neutrophil	movement speed	$speed_n$	$N(10, 1)$	$\mu m/min$	[3]
neutrophil	life span	$life\_span(n)$	$N(2160, 240)$	minutes	[9]

Descriptions and values for state variable values with literature citations, organized by cell type (with global variables indicated as such). $N(\mu, \sigma)$ indicates values taken from a normal distribution with mean μ and standard deviation σ.

Table 3: **Empiricially-determined state variables.**

State variable type	Description	Name	Value	Units
fungal spore	probability of lodging	p_{lodge}	0.05	N/A
multiple cell types	fungus detection radius	det_radius	15	μm
grid cell	diffusion rate	$diffusion\_rate$	0.8	none
epithelial cell	cytokine production factor	$cyto\_rate$	100	none
fungal hyphae	maximum iron level	$iron_{max}(f)$	2.5	proportion of iron
fungal hyphae	iron absorption rate	$iron\_abs(f)$	1	proportion of iron
fungal hyphae	iron needed for growth	$iron_{min}(f)$	0.25	proportion of iron
macrophage/neutrophil	cytokine absorption rate	$cyto\_absorb$	0.05	N/A
macrophage/neutrophil	recruitment threshold	$recr$	5	none

Descriptions and values for empirical state variable values, organized by cell type (with global variables indicated as such). $N(\mu, \sigma)$ indicates values taken from a normal distribution with mean μ and standard deviation σ.

References

[1] F. Botterel, K. Gross, O. Ibrahim-Granet, K. Khoufache, V. Escabasse, A. Coste, C. Cordonnier, E. Escudier, and S. Bretagne. Phagocytosis of Aspergillus fumigatus conidia by primary nasal epithelial cells in vitro. *BMC Microbiol.*, 8:97, 2008.

[2] W. W. Hope, V. Petraitis, R. Petraitiene, T. Aghamolla, J. Bacher, and T. J. Walsh. The initial 96 hours of invasive pulmonary aspergillosis: histopathology, comparative kinetics of galactomannan and $(1- > 3)$ β-d-glucan and consequences of delayed antifungal therapy. *Antimicrob. Agents Chemother.*, 54(11):4879–4886, Nov 2010.

[3] T. H. Howard. Quantification of the locomotive behavior of polymorphonuclear leukocytes in clot preparations. *Blood*, 59(5):946–951, May 1982.

[4] E. K. Manavathu, J. Cutright, and P. H. Chandrasekar. Comparative study of susceptibilities of germinated and ungerminated conidia of Aspergillus fumigatus to various antifungal agents. *J. Clin. Microbiol.*, 37(3):858–861, Mar 1999.

[5] B. Philippe, O. Ibrahim-Granet, M. C. Prevost, M. A. Gougerot-Pocidalo, M. Sanchez Perez, A. Van der Meeren, and J. P. Latge. Killing of Aspergillus fumigatus by alveolar macrophages is mediated by reactive oxidant intermediates. *Infect. Immun.*, 71(6):3034–3042, Jun 2003.

[6] J. Pollmächer and M. T. Figge. Agent-based model of human alveoli predicts chemotactic signaling by epithelial cells during early Aspergillus fumigatus infection. *PLoS ONE*, 9(10):e111630, 2014.

[7] J. H. Rex, J. E. Bennett, J. I. Gallin, H. L. Malech, and D. A. Melnick. Normal and deficient neutrophils can cooperate to damage Aspergillus fumigatus hyphae. *J. Infect. Dis.*, 162(2):523–528, Aug 1990.

[8] M. Schrettl, O. Ibrahim-Granet, S. Droin, M. Huerre, J. P. Latge, and H. Haas. The crucial role of the Aspergillus fumigatus siderophore system in interaction with alveolar macrophages. *Microbes Infect.*, 12(12-13):1035–1041, Nov 2010.

[9] Alan Stevens, James S. Lowe, and Barbara Young. *Wheater's Basic Histopathology: A Color Atlas and Text (Wheater's Histology and Pathology).* Churchill Livingstone, London, 4 edition, 2002.

[10] J. A. Wasylnka and M. M. Moore. Uptake of Aspergillus fumigatus Conidia by phagocytic and nonphagocytic cells in vitro: quantitation using strains expressing green fluorescent protein. *Infect. Immun.*, 70(6):3156–3163, Jun 2002.

[11] J. A. Wasylnka and M. M. Moore. Aspergillus fumigatus conidia survive and germinate in acidic organelles of A549 epithelial cells. *J. Cell. Sci.*, 116(Pt 8):1579–1587, Apr 2003.
