# Supplementary material for: A computational model of invasive aspergillosis in the lung and the role of iron
Source: BMC Syst Biol. 2016 Apr 21;10:34. doi: 10.1186/s12918-016-0275-2 (PMC4839115; doi:10.1186/s12918-016-0275-2)
Supplement: Additional file 3 — Experimental design and results of parameter sensitivity analysis. Parameters not validated by literature are altered to 10 %, 50 %, and 200 % of baseline values in order to determine the effect of each (one at a time). Relevant model dynamics are presented under both normal and neutropenic conditions (in.pdf format). (PDF 186 kb) [file 12918_2016_275_MOESM3_ESM.pdf]

Experimental design

Sensitivity analysis was performed by fixing an initial inoculum of 100 spores and then varying non-validated parameters one at a time from base levels. Base levels were determined empirically as those that gave reasonable results with respect to laboratory data. In the following table, each parameter (with one exception) was adjusted to 10%, 50%, and 200% of its baseline value (baseline values are given in the table). Each setting was run 40 times in order to obtain reliable results; data in the paper show the mean over these runs with error bars representing one standard deviation.

Table 1: **Parameter values for sensitivity analysis.**

State variable type	Description	Name	Base level	Units
fungal spore	probability of lodging	p_{lodge}	0.05	N/A
multiple cell types	fungus detection radius	det_radius	15	μm
grid cell	diffusion rate	$diffusion\_rate$	0.5	none
epithelial cell	cytokine production factor	$cyto\_rate$	100	none
fungal hyphae	maximum iron level	$iron_{max}(f)$	2.5	proportion of iron
fungal hyphae	iron absorption rate	$iron\_abs(f)$	0.5	proportion of iron
fungal hyphae	iron needed for growth	$iron_{min}(f)$	0.25	proportion of iron
macrophage/neutrophil	cytokine absorption rate	$cyto\_absorb$	0.05	N/A
macrophage/neutrophil	recruitment threshold	$recr$	5	none

Baseline values for parameter sensitivity analysis. Parameters were varied one at a time. Each was set to 10%, 50%, and 200% of the baseline value given here with the exception of the fungus detection radius, which was set to $12\mu m$, $15\mu m$, and $18\mu m$.

Results from healthy simulation

Probability of spore lodging

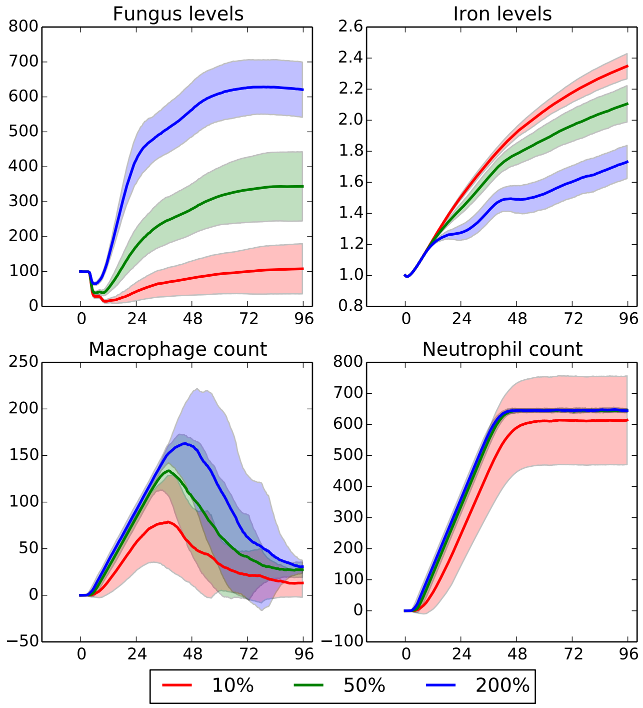

Fungus detection radius

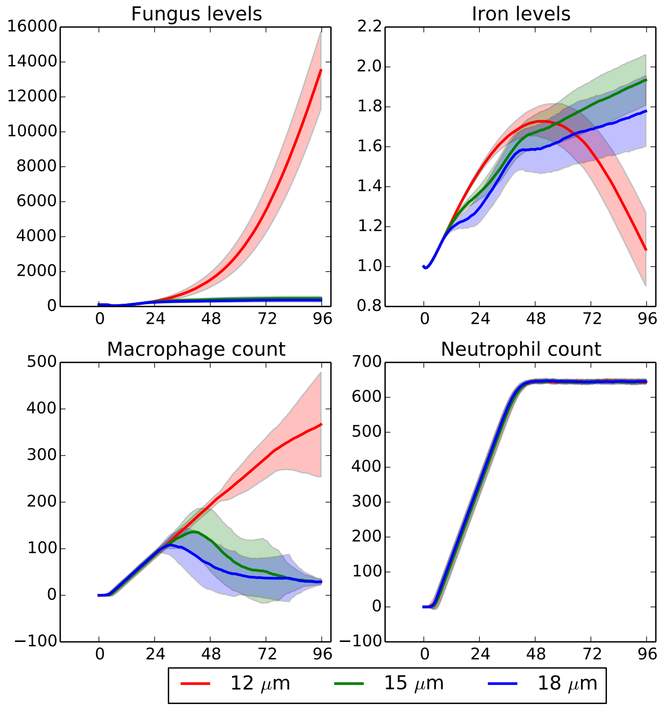

Diffusion rate

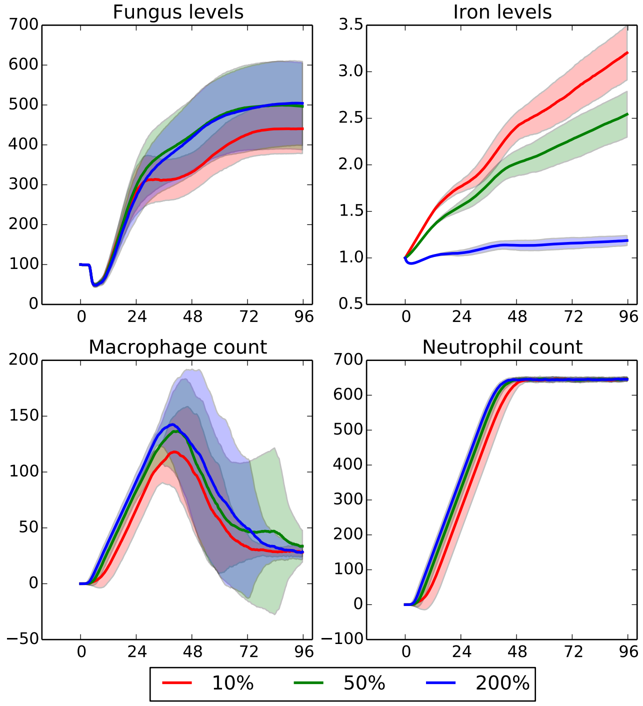

Cytokine production factor

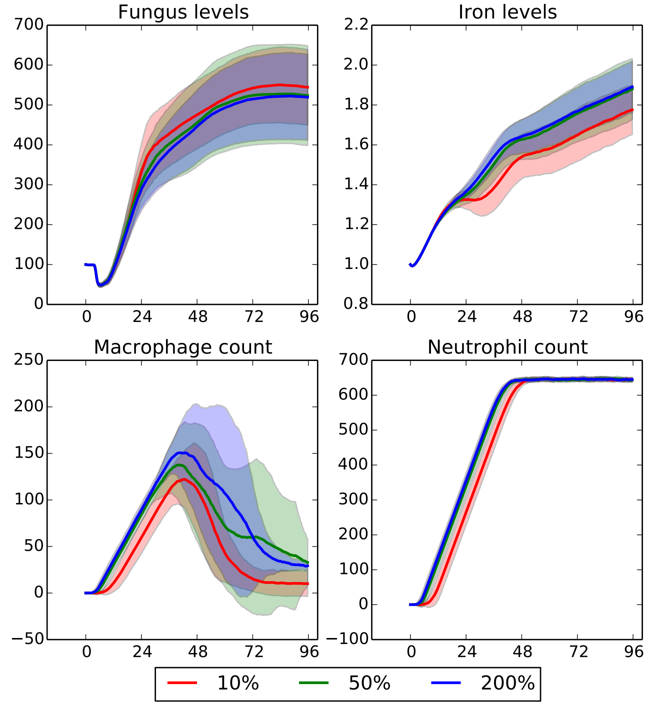

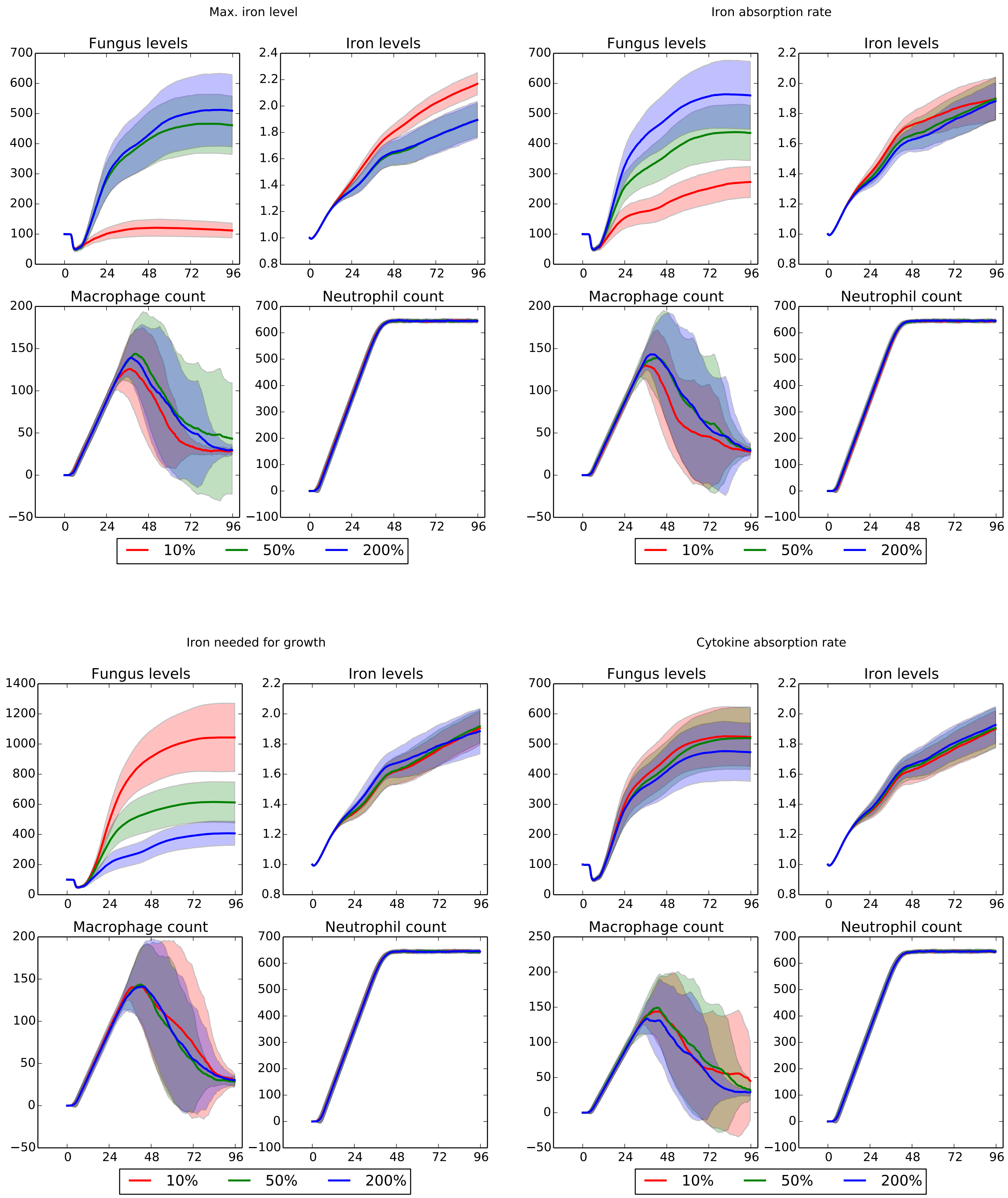

Max. iron level
Fungus levels
Iron levels
Macrophage count
Neutrophil count
10%
50%
200%
Iron absorption rate
Fungus levels
Iron levels
Macrophage count
Neutrophil count
10%
50%
200%
Iron needed for growth
Fungus levels
Iron levels
Macrophage count
Neutrophil count
10%
50%
200%
Cytokine absorption rate
Fungus levels
Iron levels
Macrophage count
Neutrophil count
10%
50%
200%

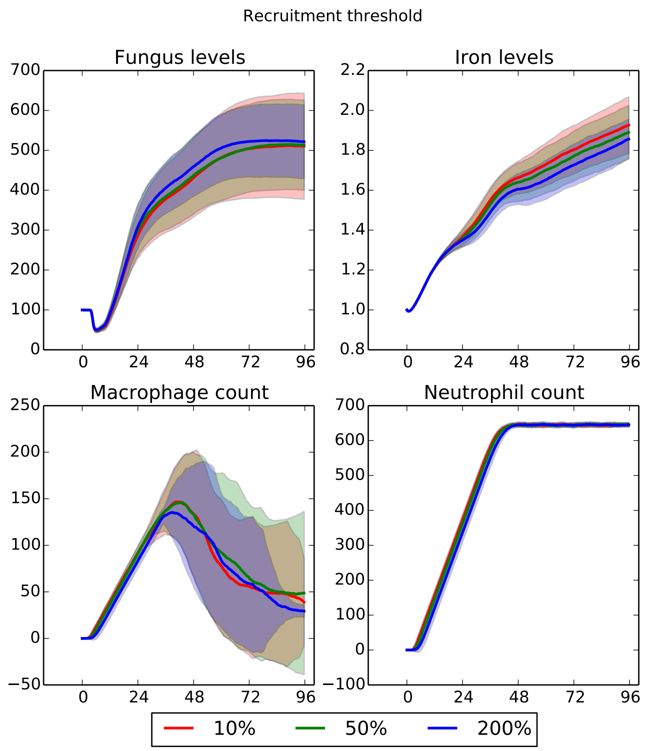
Recruitment threshold
Fungus levels
Iron levels
Macrophage count
Neutrophil count
0
24
48
72
96
10%
50%
200%

Results from neutropenic simulation

Probability of spore lodging

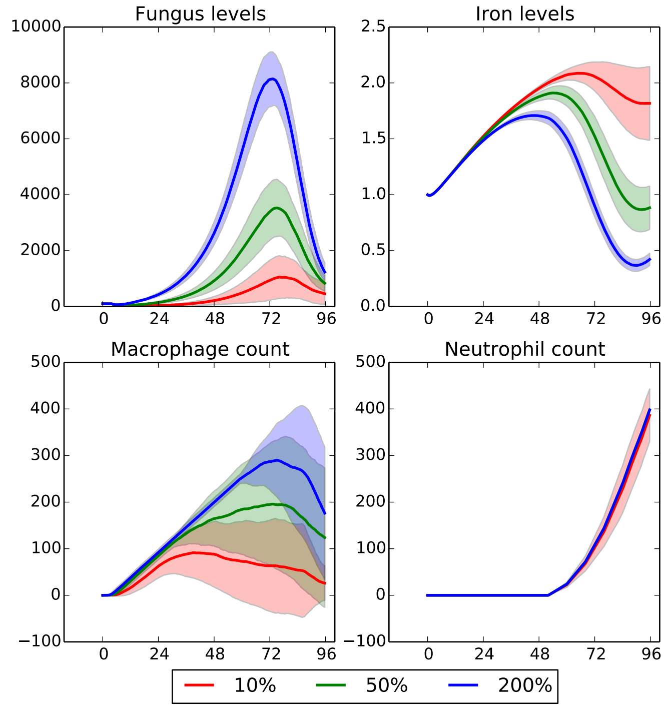

Fungus detection radius

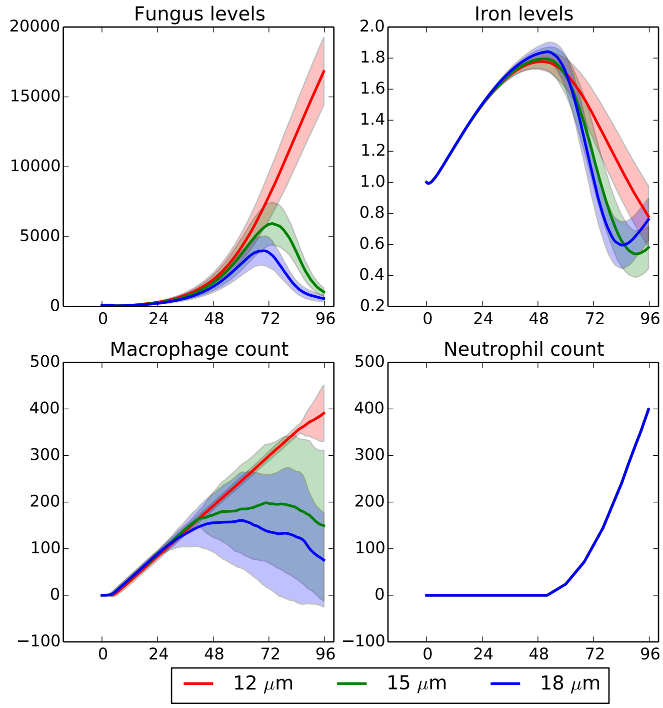

Diffusion rate

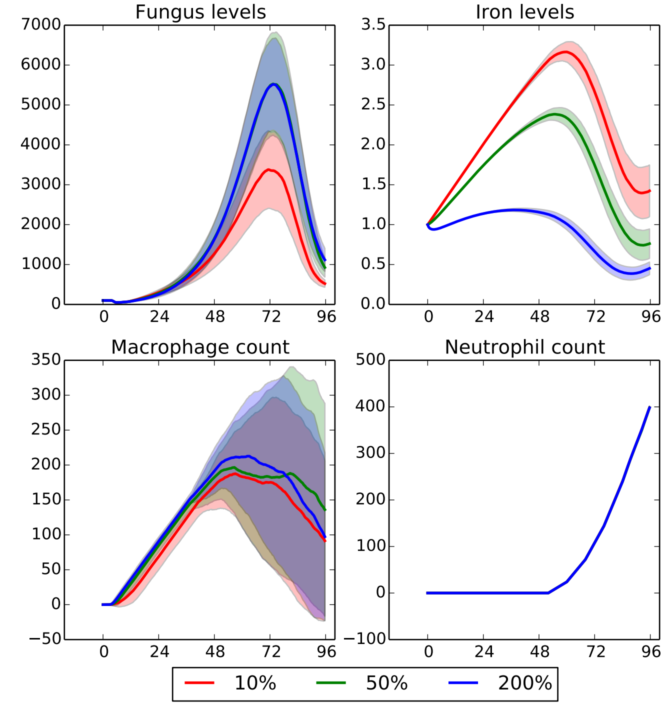

Cytokine production factor

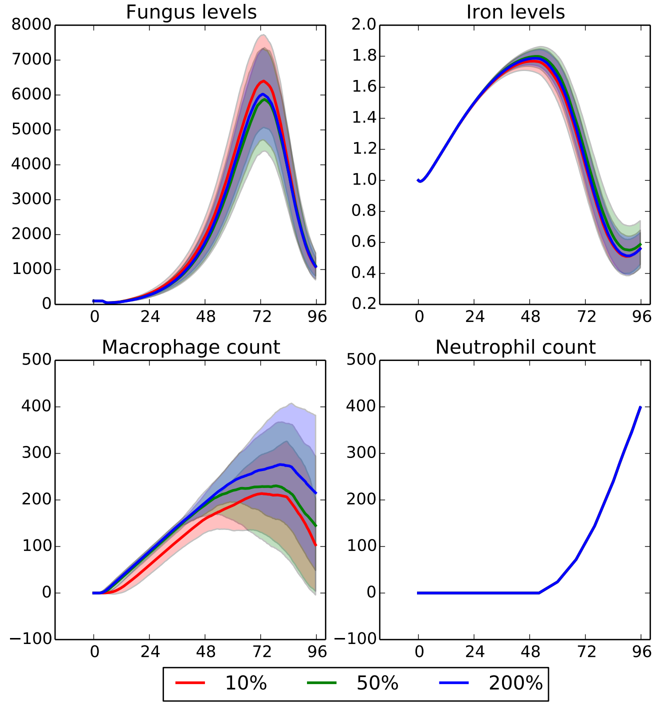

Max. iron level

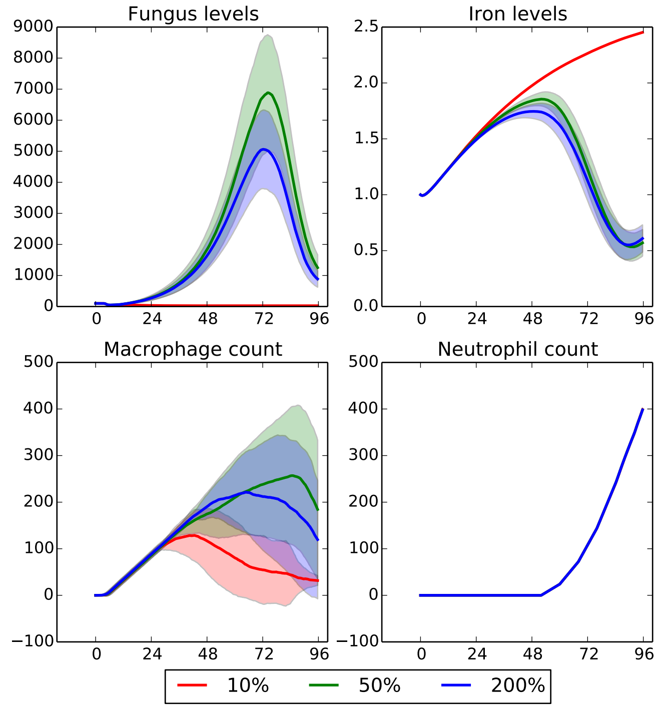

Iron absorption rate

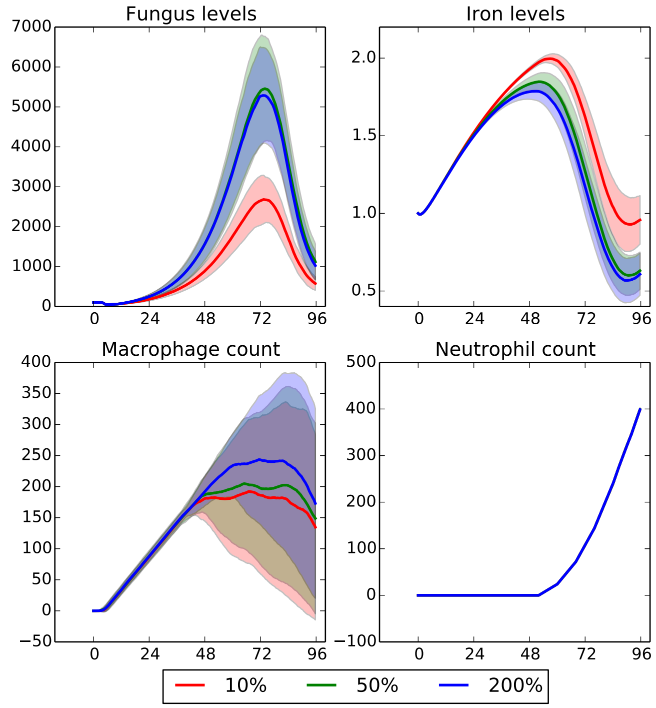

Iron needed for growth

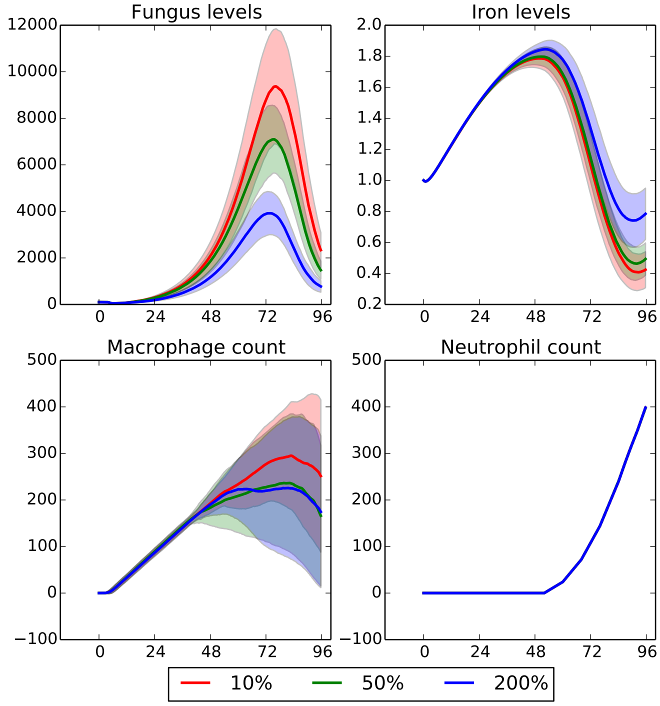

Cytokine absorption rate

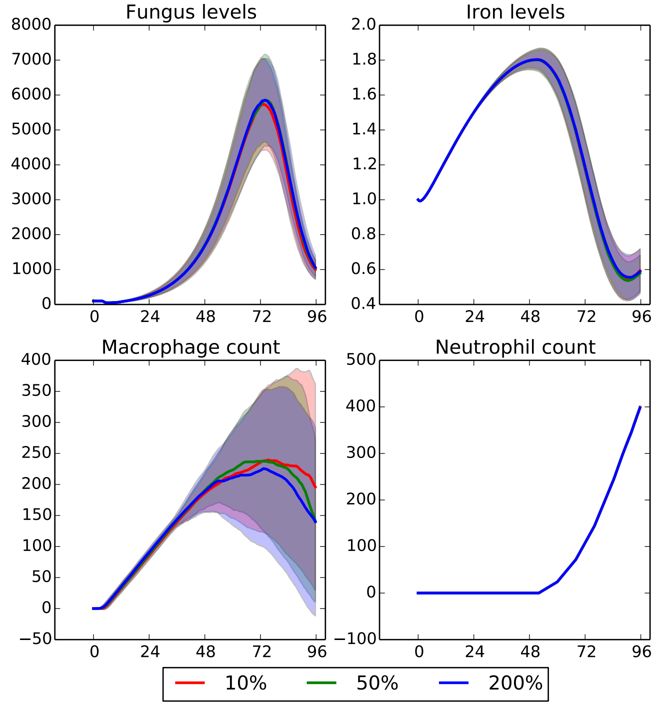

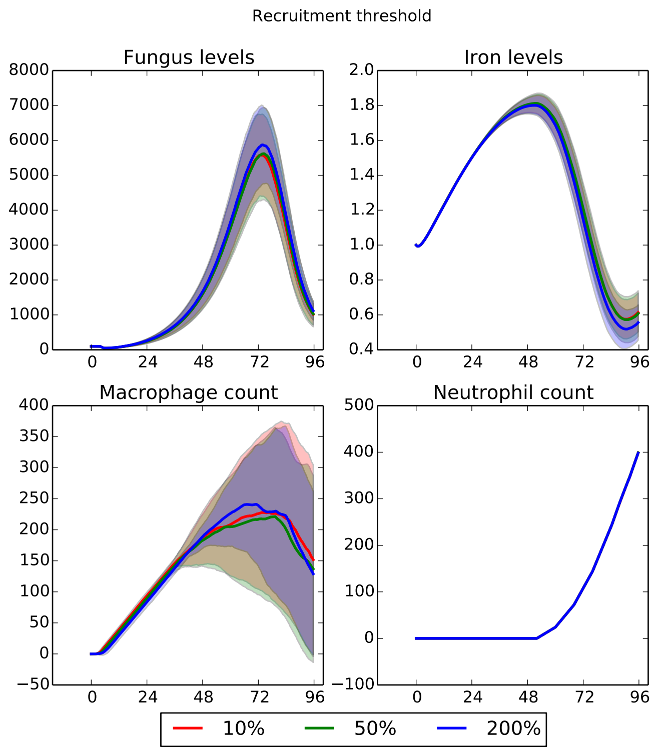
Recruitment threshold
Fungus levels
Iron levels
Macrophage count
Neutrophil count
0
24
48
72
96
8000
7000
6000
5000
4000
3000
2000
1000
2.0
1.8
1.6
1.4
1.2
1.0
0.8
0.6
0.4
400
350
300
250
200
150
100
50
−50
500
−100
10%
50%
200%
